# Persons Evaluated for 2019 Novel Coronavirus — United States, January 2020

**DOI:** 10.15585/mmwr.mm6906e1

**Published:** 2020-02-14

**Authors:** Kristina L. Bajema, Alexandra M. Oster, Olivia L. McGovern, Stephen Lindstrom, Mark R. Stenger, Tara C. Anderson, Cheryl Isenhour,, Kevin R. Clarke, Mary E. Evans, Victoria T. Chu, Holly M. Biggs, Hannah L. Kirking, Susan I. Gerber, Aron J. Hall, Alicia M. Fry, Sara E. Oliver, 2019-nCoV Persons Under Investigation Team, 2019-CoV Persons Under Investigation Team, Glen Abedi, William Bower, Kevin Chatham-Stephens, Laura Conklin, Laura Cooley, Margaret Cortese, Aaron Curns, Kathleen Dooling, Runa Gokhale, Jeremy Gold, Gavin Grant, Julie Gutman, Elisabeth Hesse, Shifaq Kamili, Lindsay Kim, Robert Kirkcaldy, Emily Koumans, Stephanie Kujawski, Gayle Langley, Joana Lively, Xiaoyan Lu, Brian Lynch, Sheryl Lyss, Lakshmi Malapati, Michael Martin, Sarah Mbaeyi, Paul McClung, Claire Midgley, Maureen Miller, Michelle Morales, Janna' Murray, Amy Parker Fiebelkorn, Manisha Patel, Georgina Peacock, Taran Pierce, Brian Rha, Senthilkumar Sakthivel, Eileen Schneider, David A. Siegel, Brittany Sunshine, Megan Wallace, Lijuan Wang, John Watson, Brett Whitaker, Anna Yousaf

**Affiliations:** ^1^Epidemic Intelligence Service, CDC; ^2^Division of Bacterial Diseases, National Center for Immunization and Respiratory Diseases, CDC; ^3^Division of HIV/AIDS Prevention, National Center for HIV/AIDS, Viral Hepatitis, STD, and TB Prevention, CDC; ^4^Division of Viral Diseases, National Center for Immunization and Respiratory Diseases, CDC; ^5^Division of STD Prevention, National Center for HIV/AIDS, Viral Hepatitis, STD, and TB Prevention, CDC; ^6^Division of Health Informatics and Surveillance, Center for Surveillance, Epidemiology, and Laboratory Services, CDC; ^7^Division of Global Health Protection, Center for Global Health, CDC; ^8^Division of Overdose Prevention, National Center for Injury Prevention and Control, CDC; ^9^Influenza Division, National Center for Immunization and Respiratory Diseases, CDC.; National Center for Immunization and Respiratory Diseases, CDC; National Center for Emerging and Zoonotic Infectious Diseases, CDC; National Center on Birth Defects and Developmental Disabilities, CDC; Center for Global Health, CDC; National Center for HIV/AIDS, Viral Hepatitis, STD, and TB Prevention, CDC; National Center for Immunization and Respiratory Diseases, CDC; National Center for Immunization and Respiratory Diseases, CDC; , National Center for Immunization and Respiratory Diseases, CDC; National Center for Emerging and Zoonotic Infectious Diseases, CDC; National Center for Emerging and Zoonotic Infectious Diseases, CDC; Center for Global Health, CDC; Center for Global Health, CDC; National Center for Emerging and Zoonotic Infectious Diseases, CDC; National Center for Immunization and Respiratory Diseases, CDC; National Center for Immunization and Respiratory Diseases, CDC; National Center for HIV/AIDS, Viral Hepatitis, STD, and TB Prevention, CDC; National Center for Chronic Disease Prevention and Health Promotion, CDC; National Center for Immunization and Respiratory Diseases, CDC; National Center for Immunization and Respiratory Diseases, CDC; National Center for Immunization and Respiratory Diseases, CDC; National Center for Immunization and Respiratory Diseases, CDC; , National Center for Immunization and Respiratory Diseases, CDC; National Center for HIV/AIDS, Viral Hepatitis, STD, and TB Prevention, CDC; National Center for Immunization and Respiratory Diseases, CDC; National Center for HIV/AIDS, Viral Hepatitis, STD, and TB Prevention, CDC; National Center for Immunization and Respiratory Diseases, CDC; National Center for HIV/AIDS, Viral Hepatitis, STD, and TB Prevention, CDC; National Center for Immunization and Respiratory Diseases, CDC; National Center for Chronic Disease Prevention and Health Promotion, CDC; Center for Global Health, CDC; National Center for Immunization and Respiratory Diseases, CDC; National Center for Immunization and Respiratory Diseases, CDC; National Center for Immunization and Respiratory Diseases, CDC; National Center for Birth Defects and Developmental Disabilities, CDC; National Center for Emerging and Zoonotic Infectious Diseases, CDC; National Center for Immunization and Respiratory Diseases, CDC; National Center for Immunization and Respiratory Diseases, CDC; National Center for Immunization and Respiratory Diseases, CDC; National Center for Chronic Disease Prevention and Health Promotion, CDC; , National Center for Emerging and Zoonotic Infectious Diseases, CDC; National Center for Immunization and Respiratory Diseases, CDC; National Center for Immunization and Respiratory Diseases, CDC; National Center for Immunization and Respiratory Diseases, CDC; National Center for Immunization and Respiratory Diseases, CDC; National Center for Immunization and Respiratory Diseases, CDC.

*On February 7, 2020, this report was posted online as an *MMWR *Early Release.*

In December 2019, a cluster of cases of pneumonia emerged in Wuhan City in central China’s Hubei Province. Genetic sequencing of isolates obtained from patients with pneumonia identified a novel coronavirus (2019-nCoV) as the etiology (*1*). As of February 4, 2020, approximately 20,000 confirmed cases had been identified in China and an additional 159 confirmed cases in 23 other countries, including 11 in the United States (*2*,*3*). On January 17, CDC and the U.S. Department of Homeland Security’s Customs and Border Protection began health screenings at U.S. airports to identify ill travelers returning from Wuhan City (*4*). CDC activated its Emergency Operations Center on January 21 and formalized a process for inquiries regarding persons suspected of having 2019-nCoV infection (*2*). As of January 31, 2020, CDC had responded to clinical inquiries from public health officials and health care providers to assist in evaluating approximately 650 persons thought to be at risk for 2019-nCoV infection. Guided by CDC criteria for the evaluation of persons under investigation (PUIs) (*5*), 210 symptomatic persons were tested for 2019-nCoV; among these persons, 148 (70%) had travel-related risk only, 42 (20%) had close contact with an ill laboratory-confirmed 2019-nCoV patient or PUI, and 18 (9%) had both travel- and contact-related risks. Eleven of these persons had laboratory-confirmed 2019-nCoV infection. Recognizing persons at risk for 2019-nCoV is critical to identifying cases and preventing further transmission. Health care providers should remain vigilant and adhere to recommended infection prevention and control practices when evaluating patients for possible 2019-nCoV infection (*6*). Providers should consult with their local and state health departments when assessing not only ill travelers from 2019-nCoV-affected countries but also ill persons who have been in close contact with patients with laboratory-confirmed 2019-nCoV infection in the United States.

As part of CDC’s Emergency Operations Center activation, CDC personnel assist state and local health departments with the evaluation of 2019-nCoV PUIs. Public health laboratories were not yet conducting 2019-nCoV testing during the period covered by this report, while awaiting Food and Drug Administration emergency use authorization for the test. (The authorization occurred on February 4*). Therefore, all testing was conducted at CDC. A call center was staffed by a team of physicians and nurses 24 hours per day. During January 17–31, criteria used to determine whether a person was considered to be a PUI included presence of fever and symptoms of lower respiratory tract illness (e.g., cough or difficulty breathing) in addition to epidemiologic risk. Epidemiologic risk factors included history of travel from Wuhan City, close contact with a patient with laboratory-confirmed 2019-nCoV infection, or close contact with an ill PUI. Given the evolving understanding of 2019-nCoV epidemiology, testing was recommended for some persons who did not strictly meet the PUI definition, based on clinical discretion. For clinical inquiries that resulted in 2019-nCoV testing, real-time reverse transcription polymerase chain reaction testing was conducted at CDC using methods developed specifically to detect 2019-nCoV (*7*).

For this report, CDC reviewed inquiries regarding potential 2019-nCoV PUIs received by CDC through January 31, 2020, from state and local health departments, health care providers, and airport health screening personnel. Information was compiled from call logs and PUI forms to assess source of inquiry, PUI demographic data (including age and sex), clinical information, epidemiologic risk factors, and 2019-nCoV test results. To allow for delays in specimen shipping and testing, data for PUIs for whom an initial inquiry was received during January 2020 were collected through February 4, 2020.

During January 2020, approximately 30 CDC physicians and nurses responded to inquiries regarding approximately 650 persons. Testing was recommended for 256 persons (Figure) across 34 jurisdictions (the jurisdictions included states, the District of Columbia, Puerto Rico, and the U.S. Virgin Islands) and was completed for 210 persons. Testing of PUIs was not always performed because alternative diagnoses were made, or symptoms resolved. Among inquiries resulting in testing, six (3%) persons were identified through airport screening, 178 (85%) in a health care setting, and 26 (12%) through contact tracing (Table). Among 178 persons identified in a health care setting, the type of setting was reported for 125 (70%), including 79 (63%) who were evaluated at an emergency department or hospital, 22 (18%) at a student clinic, and 24 (19%) in other outpatient care settings. A total of 115 (55%) persons tested were male, and median age was 29 years (interquartile range = 21–49 years). Seventeen (8%) were health care workers, and 48 of 129 persons with available information were reported to be college students.

**FIGURE:**
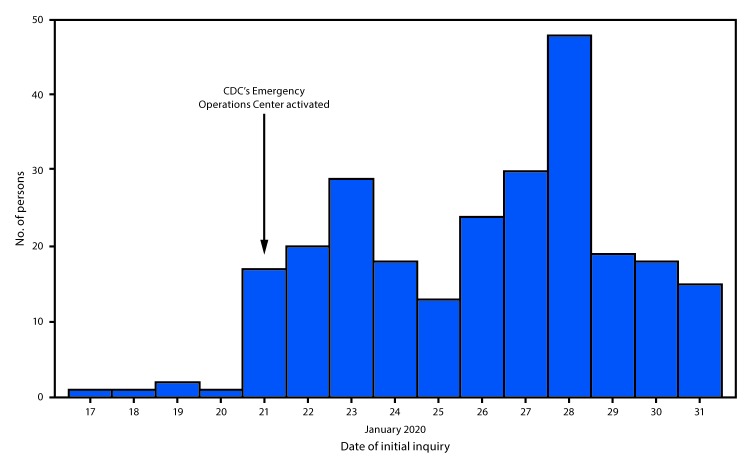
Number of persons for whom 2019 novel coronavirus (2019-nCoV) testing was recommended, by date of initial inquiry (N = 256) — United States, January 2020*^,†^ <Fig_Large></Fig_Large> * Confirmed cases were reported as of January 31, 2020. ^†^ Public announcements of a confirmed 2019-nCoV case in the United States were made on the following dates: Jan 21, Jan 24, Jan 26, Jan 27 (two cases), Jan 30, and Jan 31.

**TABLE:** Clinical characteristics and epidemiologic risk factors among persons tested for 2019 novel coronavirus (2019-nCoV) infection (N = 210) — United States, January 2020

Characteristic	Completed 2019-nCoV testing No.* (%)
**Demographics**	
**Age group (yrs), median (IQR)**	29 (21–49)
<5	10 (5)
5–17	8 (4)
18–49	138 (66)
50–64	46 (22)
≥65	4 (2)
**Male sex**	115 (55)
**Clinical features**	
**Signs or symptoms**	
Subjective fever or measured temperature ≥100.4°F (≥38.0°C)	143 (68)
Cough or shortness of breath	189 (90)
**Clinical Course**	
Hospitalized	42 (20)
Admitted to ICU	4 (2)
Died^†^	1 (<1)
**Setting where patient identified**	
Airport screening	6 (3)
Health care setting	178 (85)
Contact tracing^§^	26 (12)
**Epidemiologic risk category**	
Travel from China^¶^	148 (70)
Close contact with an ill laboratory-confirmed 2019-nCoV patient or a PUI in the United States**	42 (20)
Travel from China and close contact identified^††^	18 (9)
Other risk^§§^	2 (<1)

**Abbreviations:** ICU = intensive care unit; IQR = interquartile range; PUI = person under investigation.

* Numbers might not sum to total because of missing data.

^†^ For this person, testing was negative for 2019-nCoV, and an alternative cause of death was established.

^§^ Additional persons who were being followed through contact tracing but initially sought treatment at a health care setting are not included in this category.

^¶^ Includes 113 persons who traveled from Wuhan City and 35 who traveled from areas of China outside Wuhan within 14 days of symptom onset.

** Includes 33 persons who were close contacts of an ill laboratory-confirmed 2019-nCoV patient and nine who were close contacts of PUIs. All contacts occurred within 14 days of symptom onset.

^††^ Includes four persons who traveled from Wuhan City and were close contacts of an ill laboratory-confirmed 2019-nCoV patient, 11 who traveled from Wuhan City and were close contacts of PUIs, and three who traveled from China and were close contacts of a PUI.

^§§^ Had possible contact with a laboratory-confirmed 2019-nCoV patient and were therefore tested.

All 210 persons who were tested were symptomatic: 143 (68%) had subjective fever or a measured temperature ≥100.4°F (≥38°C), and 189 (90%) had cough or shortness of breath. Upper respiratory tract symptoms (i.e., sore throat, rhinorrhea, or congestion) were common and were present in nine persons who did not have cough or shortness of breath. Thirty persons were reported to test positive for another respiratory viral pathogen, including influenza or respiratory syncytial virus. Forty-two (20%) patients were hospitalized, and four (2%) were admitted to an intensive care unit. One patient was deceased at the time of notification; testing for this person was negative, and an alternative cause of death was established. Travel-related risk was identified for 148 (70%) persons, 42 (20%) had close contact with ill patients with laboratory-confirmed 2019-nCoV infection or PUIs, 18 (9%) had both travel- and contact-related risks, and two (<1%) had possible contact with a laboratory-confirmed 2019-nCoV patient and were therefore tested.

Among the 210 persons tested, 11 (5%) were found to have 2019-nCoV infection. Nine of these persons had traveled to Wuhan City; two persons had not traveled but had been in close contact with patients with laboratory-confirmed 2019-nCoV in the United States. All were symptomatic with fever (subjective or measured) or cough.

## Discussion

Quickly identifying persons at risk for 2019-nCoV is critical to slowing the potential spread of 2019-nCoV in the United States. This report describes CDC’s current approach to facilitating recommended diagnostic testing of persons who might have 2019-nCoV infection. In response to the emergence of 2019-nCoV in China during a time of rapidly evolving understanding of the epidemiology and clinical presentation of 2019-nCoV infection, CDC has provided consultation regarding persons suspected of being at risk for 2019-nCoV to public health officials and health care providers throughout the United States.

Epidemiologic risk factors among the 210 persons tested for 2019-nCoV were not limited to travel: 20% of PUIs tested had not recently traveled to China but reported close contact with a person being evaluated for 2019-nCoV infection. Because person-to-person transmission is expected to continue, and as further travel restrictions are implemented, it is likely that the proportion of PUIs with such contact risk in the United States will increase among all persons evaluated for 2019-nCoV.

CDC mobilized early in the response and state and local health departments similarly increased capacity to provide clinical consultation regarding 2019-nCoV. The collection of clinical and epidemiologic data that described characteristics of persons tested for 2019-nCoV helped to inform changes to criteria for PUI evaluation.

On January 31, 2020, CDC published updated PUI guidance (*8*) in response to the evolving global epidemiology of 2019-nCoV, including the rapid geographic expansion and documentation of person-to-person transmission (*9*). Updated guidance emphasizes 2019-nCoV testing for symptomatic persons in close contact with patients with laboratory-confirmed 2019-nCoV infection, persons returning from Hubei province in addition to Wuhan City, and persons from mainland China requiring hospitalization because of fever and lower respiratory tract illness. Additional refinements to this approach likely will be needed in the future as understanding of 2019-nCoV epidemiology continues to improve.

The findings in this report are subject to at least three limitations. First, the number of clinical inquiries received by CDC does not represent all inquiries received by health departments. Second, because the primary objective of this effort was to guide a timely public health response, some clinical and epidemiologic risk factor data might be incomplete. Finally, given the limited available epidemiologic information early in the outbreak, to provide some latitude for clinical decision-making regarding diagnostic testing, the PUI definition was not strictly applied in all cases.

A coordinated national effort to diagnose 2019-nCoV among persons at high risk for infection is important to facilitate appropriate management and limit transmission in the United States. CDC’s website provides guidance for health care professionals on evaluating persons for 2019-nCoV (*10*). Clinicians should maintain a high index of suspicion for possible 2019-nCoV illness not only among persons with fever and lower respiratory tract illness who report travel from China in the past 14 days but also symptomatic persons who have had close contact with patients with laboratory-confirmed 2019-nCoV infection. Clinicians should consult their local and state health departments when they suspect possible 2019-nCoV illness to facilitate diagnosis and aid prevention efforts.

**Summary**

**What is already known about this topic?**

During a 2020 outbreak of novel coronavirus (2019-nCoV) infection, CDC provided consultation to public health officials and health care providers evaluating persons at risk for 2019-nCoV infection.

**What is added by this report?**

During January 2020, CDC responded to clinical inquiries regarding approximately 650 persons in the United States and tested 210 for 2019-nCoV, one fifth of whom reported no recent travel-related risk but had close contact with a 2019-nCoV patient or a person under investigation for 2019-nCoV in the United States.

**What are the implications for public health practice?**

Health care providers should remain vigilant regarding possible 2019-nCoV exposure not only among returning travelers, but also among persons in close contact with 2019-nCoV patients in the United States.

## Footnotes

* https://www.fda.gov/news-events/press-announcements/fda-takes-significant-step-coronavirus-response-efforts-issues-emergency-use-authorization-first.
